# Trends in hospital administrative costs: urban–rural disparities, barriers, and reduction strategies

**DOI:** 10.1093/haschl/qxaf149

**Published:** 2025-08-08

**Authors:** Lauree Handlon, Kit Simpson, Larry Leaming, Dunc Williams

**Affiliations:** Department of Healthcare Leadership and Management, College of Health Professions, Medical University of South Carolina, Charleston, South Carolina 29425; Department of Healthcare Leadership and Management, College of Health Professions, Medical University of South Carolina, Charleston, South Carolina 29425; Department of Healthcare Leadership and Management, College of Health Professions, Medical University of South Carolina, Charleston, South Carolina 29425; Department of Healthcare Leadership and Management, College of Health Professions, Medical University of South Carolina, Charleston, South Carolina 29425

**Keywords:** hospital administrative costs, salary expenses, A&G

## Abstract

**Introduction:**

Administrative expenses represent a growing portion of hospital costs in the United States. However, the distribution of these costs remains underexamined. This study examines trends in administrative and general (A&G) spending among hospitals and evaluates the broader impact on expenses.

**Methods:**

We conducted a sequential explanatory mixed methods study, analyzing Medicare cost report data from 2011 to 2022 for all US short-term acute care hospitals. Quantitative analysis focused on A&G salary expenses and total A&G costs as a percentage of overall hospital expenditures. Findings were supplemented with qualitative interviews with hospital executives to contextualize observed trends.

**Results:**

While urban hospitals reported higher total A&G expenses, rural hospitals consistently allocated a larger proportion of spending to A&G salaries, 18% more on average. Across all hospital types, A&G salary costs declined as a share of total expenses while total administrative costs increased, reflecting a shift toward nonsalary drivers.

**Conclusions:**

Rising administrative costs are primarily driven by systemic and structural demands, rather than salaries. Addressing these challenges will require targeted policy responses that simplify processes and support the unique needs of hospitals with limited resources, particularly in rural communities. Such reforms may enhance financial resilience and promote sustainable access to care.

## Teaser

Administrative spending constitutes a substantial and growing share of hospital expenditures, yet the underlying cost dynamics remain insufficiently understood. Using national cost report data from 2011 to 2022, this study finds that while total administrative expenses have grown, administrative salary costs have declined as a share of total hospital spending. Rural hospitals, however, continue to carry a disproportionate burden, spending 18% more of their total expenses on administrative salaries than urban hospitals. These findings challenge the belief that administrative salaries increasingly contribute to administrative cost growth. Instead, the findings highlight broader pressures, including regulatory complexity and operational demands. A clearer understanding of these cost trends is critical to informing policy reforms aimed at reducing administrative burden and supporting the long-term financial stability of hospitals, particularly those serving rural communities.

Key Points

- Urban hospitals incurred higher total administrative costs, but rural hospitals allocated more of their administrative spending to salaries.
- On average, rural hospitals expended 18% more on administrative salaries than urban hospitals, highlighting structural cost challenges in rural healthcare.
- Although administrative salaries often draw public attention, the data show that administrative and general salary costs have declined as a share of hospital total administrative costs across all facility types.

## Introduction

Administrative and general (A&G) costs represent a growing but often misconstrued financial burden in US hospitals. As healthcare costs continue to rise, hospitals, particularly those in rural areas, face increasing financial pressures driven by administrative complexity. Research estimates that administrative expenses constitute 15% to 30% of US healthcare expenditures.^1^ While urban hospitals may achieve greater economies of scale, allowing them to absorb some of these costs, rural hospitals often struggle with fewer resources and higher administrative costs.

Public dialogue on hospital administration, particularly with A&G costs, often centers on executive compensation, with media coverage frequently emphasizing the salaries of highly compensated hospital leaders.^2-4^ This emphasis may contribute to the perception that administrative salaries, broadly defined, represent a growing share of healthcare expenditures. However, data on A&G expenses may suggest a more complex reality.

Existing literature highlights the growing share of hospital budgets allocated to administrative functions. A&G costs encompass a wide range of nonclinical expenses, including compliance, legal, and accounting services, as well as various expenses related to facility administrative services not already reported under other service cost centers.^5^ Administrative costs in US hospitals have steadily increased since the early 2000s.^6^ Exacerbated by the COVID-19 pandemic, heightened regulatory demands, and fueled by the complex and disjointed nature of the US payer system, costs continue to rise.^7-9^

Hospitals must navigate diverse payer rules, engage in extensive documentation for reimbursement, and dedicate significant resources to compliance-related activities.^10^ The costly impact of manual administrative processes requires substantial labor investments.^11^ As a result, administrative inefficiencies contribute to financial strain, particularly for hospitals with restricted resources. Despite the pressing nature of this issue, there is a limited understanding of how A&G costs impact hospital financial sustainability at the facility level.

Previous studies have linked administrative inefficiencies to rising healthcare costs; however, facility-level analysis remains limited. Addressing administrative complexity is crucial for ensuring hospital financial sustainability and maintaining high-quality patient care. Policymakers and hospital administrators must develop strategies that minimize administrative inefficiencies without compromising compliance and operational integrity, particularly in rural hospitals facing financial challenges and potential closure.^12,13^ By identifying cost reduction opportunities, this study offers actionable insights for stakeholders across the healthcare sector concerned with the viability of rural healthcare facilities.

By leveraging data from the Centers for Medicare & Medicaid Services’ Healthcare Cost Reporting Information System (HCRIS), we explored empirical insights into the financial impact of administrative complexities on hospitals. The data revealed trends in A&G expenses among US hospitals from 2011 to 2022, as well as differences between urban and rural areas. Additionally, qualitative perceptions from hospital executives further contextualized the barriers and opportunities associated with cost drivers and potential mitigation strategies.

The following research questions provided directions for this study:

1. Has A&G spending (salary and total) changed between 2011 and 2022?
2. Do A&G costs differ by rurality?
3. What costs are reported in the A&G nonsalary category in the cost report?
4. What barriers and opportunities exist to reduce A&G expenses among US hospitals?

These questions were the cornerstone of our investigation into cost trends related to administrative complexity and strategy exploration for cost reduction. Our insights are of significant value to policy decision-makers, offering a potential roadmap to enhance healthcare efficiency.

## Data and methods

### Identifying and integrating hospital data sources

We constructed a panel of hospital-level data for 2011-2022 using CMS HCRIS files, also known as “cost reports.”^5,14^ To measure health system affiliation, we merged cost report data with American Hospital Association Annual Survey (AHAAS) data (2017-2021) and the Agency for Healthcare Research and Quality (AHRQ) Compendium (2016-2022).^15^ Cost reports are a widely adopted source of hospital financial information because all hospitals are required to file these reports with CMS annually to receive Medicare reimbursement.

### Study sample

The study included all short-term, general acute care hospitals in the US that submitted cost reports between 2011 and 2022. The data excluded hospitals with incomplete financial data (eg, <360 days in a reporting period) or hospitals not classified as general acute care facilities. We applied the following exclusion criteria to all years within the sample to account for these 2 exclusion criteria. For example, for 2022, we began with a sample of 4595 hospitals. We excluded hospitals reporting <360 days in a reporting period (*n* = 43 in 2022, 0.9%), resulting in *n* = 4542. We then excluded all non-US states (*n* = 55 in 2022, 1.2%), resulting in *n* = 4487. Next, we excluded specialty hospitals and Indian Health Service hospitals (*n* = 63 in 2022, 1.37%), resulting in *n* = 4424.

### Study design

Our study employed a sequential explanatory mixed-methods design, which allowed us to thoroughly investigate trends in hospital A&G expenses and contextualize them through the experiences of hospital executives.^16^ Quantitatively, hospital financial and operational cost report data (2011-2022) were examined, focusing on A&G salary spending as a percentage of hospital expenditures stratified by rurality. Rurality was defined according to the criteria specified by the Federal Office of Rural Health Policy.^17^

The relationship between A&G salary spending as a percentage of total expenses and theorized covariates was fitted using generalized estimating equations. Standard errors were adjusted for hospital-level clustering. Descriptive bivariate differences between treatment and comparison hospitals were tested using Pearson's χ^2^ for categorical variables and two-sided *t*-tests for continuous variables. The threshold for statistical significance was set a priori at *P* < .05. To account for outliers, the data were Winsorized at the 1% tails of each variable's distribution.^18,19^ The unit of analysis was the hospital year.

To complement these findings, semi-structured interviews were conducted with 5 hospital executives, including 4 CEOs and 1 CFO, who were experienced in supporting cost reports. An interview guide (Supplementary Appendix A) was developed based on preidentified themes reflecting the study's primary domains of interest, including trends in A&G costs, cost drivers, barriers to reporting, opportunities for cost reduction, and reactions to quantitative data. The initial themes assisted in structuring the interviews but did not constrain the emergence of new insights. As interviews progressed, subthemes were identified inductively based on participant responses. Interviews were conducted via Microsoft Teams, recorded with consent, and transcribed verbatim.

Thematic analysis followed an iterative, exploratory process consistent with the SRQR reporting standards.^20^ After manual review and preliminary coding of each transcript, summaries of the anonymized content were entered into OpenAI ChatGPT 4.0^21^ to support theme synthesis and grouping. The AI model was not used to analyze raw transcripts and did not have access to identifiable data. The model was employed in a supplemental role to enhance thematic clarity and reduce redundancy.

Prompts used included general framing questions like, “How might the information be grouped into subcategories given the following summary statements?” and “Based on the following summary statements, do any of these subthemes appear redundant or overlapping?” The AI-generated outputs were then reviewed, validated against the original transcripts, and revised where necessary to ensure accuracy, contextual relevance, and reliability to participants’ intended meaning.

The analytic approach maintained full analyst oversight and ensured interpretive consistency, with AI tools serving as secondary aid to support thematic refinement. Integrating qualitative insights with quantitative trends provided a comprehensive understanding of financial patterns and operational challenges in managing hospital administrative costs.

### Study variables

The key outcome variables in this study measured different aspects of hospital administrative expenses, including A&G salary expenses and total A&G expenditures as percentages of total hospital expenses. Presenting A&G salary data as a proportion of total hospital expenditures rather than raw dollar amounts enabled a clearer assessment of whether administrative costs increased at a rate distinct from overall hospital spending. This approach helped determine a shifting focus in spending priorities, that is, whether hospitals are dedicating more or less of their budgets to administrative costs compared with other expenses. Additionally, expressing A&G salary expenditures as a percentage of total hospital spending enables meaningful comparisons across hospitals of different sizes. Both measures were logged because of skewed distributions. These measures provide insights into operational overhead, workforce-related administrative costs, and financial efficiency.

The control variables include rural status, critical access hospital (CAH) designation, year, length of stay (LOS), total uncompensated care and bad debt, days in net accounts receivable (Net A/R), days cash on hand (DCOH), long-term debt to capitalization ratio (LT Debt-to-Cap), commercial to Medicare revenue-to-charge ratio (estimated), and whether the hospital was part of a health system.

### Limitations

Our study has limitations. While CMS cost reports are widely used for hospital financial data because all hospitals are required to file annually with CMS to receive Medicare reimbursement, prior research has noted inconsistencies in reporting.^22^ Nonetheless, the accessibility of financial data for all hospitals receiving CMS-based reimbursement and its frequent use in research support the reliability of these data. Health system participation was determined using the AHRQ Compendium (2016-2022) and AHAAS (2017-2021), but hospitals missing from these datasets were not captured.

Furthermore, HCRIS data lacks granularity in administrative expenditures, limiting the ability to isolate cost drivers. Qualitative interviews with 5 hospital leaders provided contextual insights to address this, though findings may not fully capture hospital-level variations. Potential recall and interviewer biases exist but were mitigated through structured protocols. Despite these limitations, the findings remain robust and contribute to understanding hospital administrative costs.

## Results

### Quantitative results

#### Hospital characteristics

The dataset includes 4684 hospitals in 2011 and 4424 in 2022, with rural hospitals comprising 50.4% of the sample in 2022. Table 1 presents hospital characteristics across urban and rural settings in 2011 and 2022, highlighting significant differences in demographics, operations, and financial attributes. All bivariate associations reported in the following sections were statistically significant (*P* < .001).

**Table 1.** Unadjusted hospital characteristics, urban and rural, 2011 and 2022, with percent change.

	2011		2022		Percent Change (%)	
	Urban (*N* = 2359)	Rural (*N* = 2325)	Urban (*N* = 2193)	Rural (*N* = 2231)	Urban	Rural
**Demographic characteristics**						
Hospital beds—total	234	48^a^	249	43^a^	6.4%	−10.4%
Employee FTEs	1451	277^a^	1687	291^a^	16.3%	5.1%
Ownership type						
Not for profit	1446	1217^a^	1423	1287^a^	−1.6%	5.8%
For profit	644	294	548	214	−14.9%	−27.2%
Government—nonfederal	269	814	222	730	−17.5%	−10.3%
Critical access hospital (CAH)						
Non-CAH	2359	921^a^	2193	872^a^	−7.0%	−5.3%
CAH	0	1404	0	1359	N/A	−3.2%
Region						
Northeast	428	169^a^	376	160^a^	−12.1%	−5.3%
Midwest	516	884	471	856	−8.7%	−3.2%
South	910	887	844	832	−7.3%	−6.2%
West	505	385	502	383	−0.6%	−0.5%
Health system						
Not part of health system	2359	2325	153	690^a^	−93.5%	−70.3%
Part of health system	N/A	N/A	2040	1541	N/A	N/A
**Operational characteristics**						
Average daily census	151.5	20.2^a^	170.8	17.8^a^	12.8%	−11.9%
Length of stay	4.4	4.9^a^	4.9	5.8^a^	10.9%	18.4%
Case mix index	1.6	1.2^a^	1.9	1.5^a^	19.9%	22.0%
**Financial characteristics**						
Total expenses ($)	242 740 333	40 472 517^a^	466 180 016	68 353 201^a^	92.0%	68.9%
A&G total expenses ($)	48 038 942	6 886 955^a^	92 781 045	11 673 235^a^	93.1%	69.5%
A&G salary expenses ($)	10 384 418	2 013 449^a^	15 663 753	2 924 153^a^	50.8%	45.2%
A&G expenses to total expenses (%)	20.0	16.9^a^	21.0	17.6^a^	4.9%	4.1%
A&G salary expenses to total expenses (%)	4.4	5.4^a^	3.6	4.7^a^	−18.3%	−12.2%
Operating margin (%)	4.0	0.2^a^	1.9	0.3^a^	−52.9%	47.8%
Days in net A/R	51.4	55.9^a^	57.6	55.5	12.1%	−0.7%
Commercial-to-Medicare RCR	1.6	1.6	1.5	1.4	−10.2%	−11.2%
Total UCC and bad debt	7.4	7.9^b^	7.0	7.4^b^	−6.2%	−6.3%
Days cash on hand	124.6	136.7	127.2	177.6^a^	2.0%	29.9%
LT Deb-to-Cap (%)	38.7	32.8^a^	29.2	26.1^a^	−24.4%	−20.4%

Source: Authors’ analysis of Medicare Hospital Cost Reports from 2011 to 2022, merged with American Hospital Association Annual Survey data (2017-2021) and the AHRQ Compendium (2016-2022). Notes: Variable distributions are shown as N (categorical) and mean (continuous). Abbreviations: FTE, full-time equivalent; RCR, revenue-to-charge ratio; UCC, uncompensated care; LT Deb-to-Cap, long-term debt to capitalization ratio. “N/A” indicates that data are unavailable or the calculation could not be executed due to missing or zero values. Missing Observations: See Supplementary Appendix F. ^a^*P* < .001. ^b^*P* < .01.

#### Demographic characteristics

As anticipated, urban hospitals displayed a significantly higher mean bed capacity than their rural counterparts (249 vs 43 in 2022). However, the disparity in growth rates of bed capacity was stark, with urban hospitals increasing by 6.4% from 2011 to 2022, while rural hospitals experienced a 10.4% decline. The 2022 ownership structures also exhibited notable variations across different settings. Not-for-profit hospitals were more prevalent in urban areas (64.9% vs 57.7% rural), while government nonfederal hospitals were more common in rural areas (32.7% vs 10.2% urban). The CAH designation, exclusive to rural hospitals, applied to 60.9% of rural facilities in 2022.

#### Operational characteristics

Urban hospitals maintained a higher 2022 mean daily census (170.8 vs 17.8 rural), a difference that continued to widen over time. Urban hospitals saw a 12.8% increase, while rural hospitals experienced an 11.9% decrease from 2011 to 2022. Interestingly, the 2022 mean LOS was paradoxically higher in rural hospitals (5.8 vs 4.9 days urban), while the case mix index (CMI) was higher in urban hospitals (1.9 vs 1.5 rural). Although urban hospitals exhibited higher CMI values, which may reflect greater clinical complexity, this difference could also stem from variation in coding practices. While coding practices are intended to reflect clinical reality as accurately as possible, differences in payment structure may influence coding intensity and thereby impact CMI calculations.

#### Financial characteristics

In 2022, the mean total expenses were notably higher in urban hospitals ($466.1 M vs $68.3 M rural), and A&G total expenditures were nearly 8 times higher in urban settings ($92.8 M vs $11.7 M rural). Urban hospitals also allocated a greater share of total expenses to A&G costs (21.0% vs 17.6% rural), including higher A&G salary expenses ($15.7 million vs $2.9 million rural). Over time, total hospital and A&G costs increased in both settings; however, A&G salary expenses declined as a percentage of total hospital spending for urban hospitals, while rural hospitals maintained a higher proportion of salary-related A&G costs (4.7% vs 3.6% urban).

The change in operating margins differed over time. The margin for urban hospitals declined to 1.9 from 4.0, while the margin for rural hospitals remained critically low, from 0.2 in 2011 to 0.3 in 2022. In 2022, the DCOH was significantly higher in rural hospitals than in urban hospitals (177.6 vs 127.2 days), likely influenced by COVID-19 provider relief funds, which strengthened cash reserves, particularly in rural settings. LT Debt-to-Cap ratios were lower in 2022 for rural hospitals (26.1% vs 29.2% urban).

#### Unadjusted trends in percentages of A&G salary costs to total hospital costs

**Figure 1.**
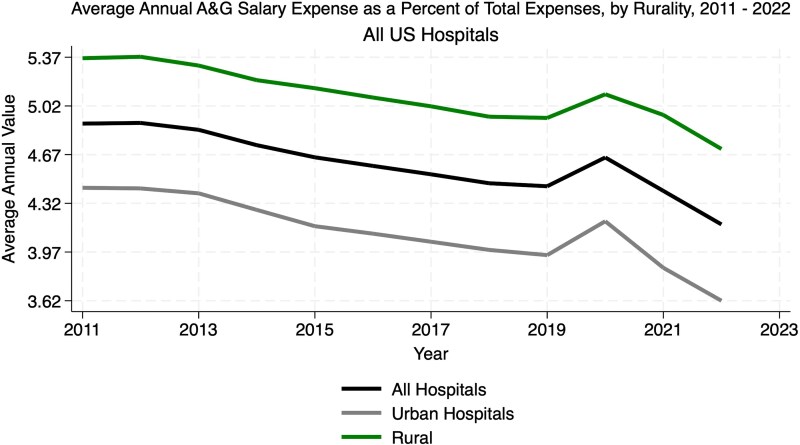
Average annual A&G salary expenses as a percent of total expenses by rurality, 2011-2022. Source: Authors’ analysis of Medicare Hospital Cost Reports from 2011 to 2022, merged with American Hospital Association Annual Survey data (2017-2021) and the AHRQ Compendium (2016-2022). Notes: The percentages represent nominal values without adjustment, offering a baseline comparison of salary expense distribution across rural and urban hospitals.

Figure 1 displays the unadjusted average A&G salary expenses as a percentage of total hospital expenses for 2011 and 2022, categorized by rurality. Urban hospitals show a decline, ending significantly lower than rural hospitals in 2022. Rural hospitals maintain higher salary expenses but also experience a decrease, with a temporary rise around 2019-2020. The overall trend for all hospitals indicates a gradual reduction in administrative salary expenses as a percentage of total costs, driven largely by urban hospital trends.

Urban hospitals consistently allocated a larger proportion of total expenses to A&G costs, reflecting broader operational scopes. Supplementary Appendix B shows that A&G’s total expenses increased across both hospital types, peaking around 2020 before declining slightly in subsequent years.

#### Adjusted trends in percentages of A&G salary costs to total hospital costs

Table 2 displays the regression-adjusted results. After controlling for CAH designation, year, LOS, total uncompensated care and bad debt, net A/R, DCOH, LT debt-to-cap, commercial-to-Medicare revenue-to-charge ratio, and health system affiliation, rural hospitals spent 18% more on A&G salaries relative to total expenses compared with urban hospitals (*P* < .001). Being a CAH in the Northeast, operating as either a for-profit or governmental not-for-profit, holding more cash on hand, billing less commercial revenue relative to Medicare, and not being part of a health system was also associated with a higher percentage of A&G salary spending relative to total spending.

**Table 2.** Estimated changes in A&G salary spending, 2011-2022.

Independent variable	Coefficient	Percentage change (%)^a^
Rural hospitals	0.166^b^	18.05
**Demographic characteristics**		
Ownership type		
For profit	0.127^b^	13.50
Government—nonfederal	0.056^b^	5.72
Critical access hospital (CAH)		
CAH	0.089^b^	9.38
Region		
Midwest	−0.227^b^	−20.35
South	−0.189^b^	−17.19
West	−0.142^b^	−13.20
Health system affiliation		
Part of health system	−0.079^b^	−7.57
**Operational characteristics**		
Length of stay	−0.004	−0.39
**Financial characteristics**		
Days in net A/R	−0.000	−0.02
Commercial-to-Medicare RCR	−0.018^c^	−1.82
Total UCC and bad debt	0.001	0.66
Days cash on hand	0.000^b^	0.12
LT deb-to-cap	0.000	0.02
Year scaled	−0.005^b^	−0.49
Intercept	1.681^b^	

Source: Authors’ analysis of Medicare Hospital Cost Reports from 2011 to 2022, merged with American Hospital Association Annual Survey data (2017-2021), and the AHRQ Compendium (2016-2022). Notes: The analysis is based on 30 621 hospital-year observations from Medicare Hospital Cost Reports (2011-2022). The trends depicted provide insights into the evolution of A&G salary expenditures across hospitals, highlighting variations by key characteristics such as rurality. Abbreviations: RCR, revenue-to-charge ratio; UCC, uncompensated; LT Deb-to-Cap, long-term debt to capitalization ratio. ^a^Because the outcome was log-transformed, we converted the estimated percentage change by 100*(exp(b)-1) in the “Percentage Change” column. ^b^*P* < .001. ^c^*P* < .01.

**Figure 2.**
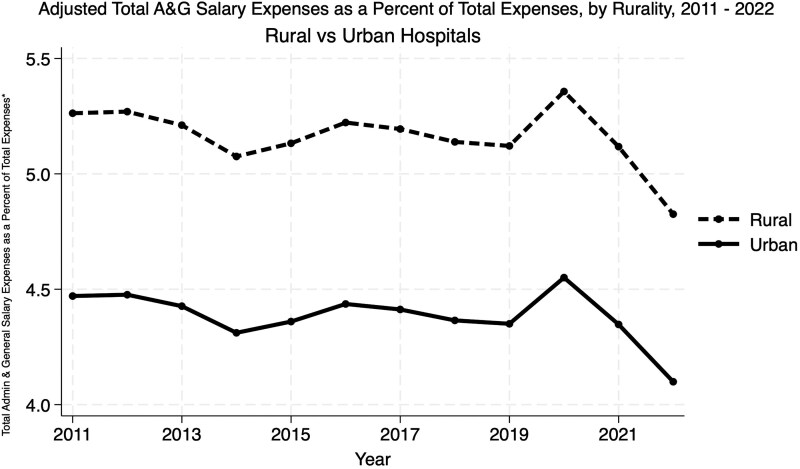
Adjusted total A&G salary expenses as a percent of total expenses, by rurality, 2011-2022. Source: Authors’ analysis of Medicare Hospital Cost Reports from 2011 to 2022, merged with American Hospital Association Annual Survey data (2017-2021) and the AHRQ Compendium (2016-2022). Notes: The trends depicted regression-adjusted insights into the evolution of A&G salary expenditures across hospitals, highlighting variations by key characteristics such as rurality.

Figure 2 illustrates that after controlling for the factors above, rural hospitals consistently allocated more total expenses to A&G salaries than urban hospitals (5.3% in 2011, 4.8% in 2022 for rural, 4.5% in 2011, and 4.1% in 2022 for urban). Overall, urban hospitals faced greater total administrative cost burdens, as shown in Supplementary Appendix C, likely reflecting more complex operations. Although both urban and rural hospitals experienced parallel upward trends in total A&G expenses as a percentage of total expenses, a persistent disparity remained, with rural hospitals consistently allocating a higher share of their budgets to administrative costs throughout the study period.

### Qualitative results

The qualitative data from the key informant interviews provided valuable context to support the quantitative findings. While the primary focus of the study was on hospital cost report data, the interviews enriched the understanding of operational dynamics behind rising A&G costs. The emerging domains from the experts reflected drivers, barriers, and reduction strategies for A&G costs, enriching the understanding of the complex factors involved. The qualitative component was designed to supplement the quantitative findings. These qualitative insights, presented in Supplementary Appendices D and E provide a widespread perspective of the challenges and opportunities in hospital administration.

#### Theme 1: key drivers of A&G cost growth

Participants cited expanding regulatory and fragmented payer requirements necessitating larger administrative teams, investments in revenue cycle management, and advanced electronic health records (EHRs) as key drivers of rising costs. One participant stated,

“Payers are increasingly finding ways to deny claims, delay payments, or underpay us. It takes more administrative resources to keep up, which means more staff, more technology, and ultimately, higher costs.”

Participants also noted that cybersecurity and IT infrastructure investments may lead to further increases in administrative expenditure.

#### Theme 2: barriers to accurate A&G cost reporting

Inconsistent hospital cost categorization and challenges meeting complex Medicare cost reporting requirements were cited as barriers to accurate A&G reporting. These barriers were particularly pronounced in resource-constrained rural hospitals, where staff often had to manage multiple roles. One participant expressed,

“Many rural hospitals can’t afford full-time financial specialists, so cost reporting falls on staff who wear multiple hats. That leads to inconsistencies in how expenses are classified.”

#### Theme 3: strategies to manage and reduce A&G costs

Strategies to reduce A&G costs include automation, heightened internal oversight, and role consolidation, supported by legislative advocacy to address payer inefficiencies. One participant stated regarding legislative advocacy against payer inefficiencies,

“We’ve been actively pushing for policy changes to reduce the administrative burden from payers, whether it's streamlining prior authorizations or enforcing timely payment rules. The current system forces us to dedicate too many resources to fighting denials and delays.”

By implementing these approaches, hospitals can streamline operations, reduce administrative burden, and reallocate resources toward patient care, thereby promoting a more efficient and sustainable healthcare system.

Urban–rural disparities in hospital operations, finances, and administrative spending from 2011 to 2022 are evident. The insights of hospital leaders further highlight challenges related to administrative complexity, cost reporting, and containment strategies. Together, the qualitative context and quantitative results provide a strong foundation for policymakers, administrators, and researchers to drive meaningful reforms across the healthcare sector.

## Discussion

Analyzing A&G salary expenses challenges the perception that administrative salaries drive healthcare cost growth. Between 2011 and 2022, administrative salaries declined as a proportion of total hospital expenditures across all hospital types. Despite rising healthcare costs, hospital administrator salaries have not fueled this growth but represent a shrinking share of total spending, underscoring a broader shift in cost dynamics.

Despite the decline, rural hospitals consistently allocate a higher proportion of expenses to A&G salary costs than their urban counterparts. The continued disparity suggests rural hospitals face structural challenges in reducing administrative salary expenses, likely due to smaller economies of scale, staffing limitations, and fewer opportunities to distribute workloads across specialized administrative teams.

In parallel, qualitative interviews with hospital executives highlighted 3 key patterns: administrative complexity, particularly in cost reporting, remains a significant burden; rising nonsalary administrative costs (eg, legal, IT, and compliance expenses) are outpacing traditional staffing costs; and current payer structures and documentation requirements significantly contribute to inefficiencies. Many executives reported that rising administrative demands from both payers and regulators are difficult to absorb, especially in resource-constrained rural settings.

### Implications for policy and practice

The quantitative and qualitative results underscore the need for practical, evidence-informed interventions. Rather than proposing comprehensive, system-wide reforms, targeted strategies are more practical, aligned with observed trends, and informed by executive experiences. The goal is not to eliminate administrative structures, but to enhance efficiency, alleviate disproportionate burdens on rural hospitals, and support sustainable operations. The following policy and practice implications reflect this targeted approach.

#### Streamlining administrative cost reporting and support for rural hospitals

Although total administrative expenses are higher at urban hospitals, rural hospitals consistently allocate a larger proportion of their operating budgets to A&G costs. The rural-urban disparity in A&G salary spending reflects deeper structural issues rather than inefficiencies. Structural factors include smaller economies of scale, fewer specialized staff, and constrained financial resources, which reduce the capacity to absorb administrative burden. Policymakers should consider offering administrative support models, such as shared service partnerships, technical assistance for cost reporting, or simplified documentation protocols tailored to the capacity of rural hospitals. Such efforts can help alleviate disproportionate administrative strain and support financial sustainability in rural healthcare settings.

#### Recognizing the shift toward nonsalary administrative burden

The data showed that while A&G salaries are declining as a share of total hospital expenses, nonsalary categories, which include, by cost report definition, legal, IT, and compliance costs, are increasing as a proportion of total hospital spend. These trends may not be fully captured in existing hospital efficiency benchmarks. Policymakers and payers should consider updating how administrative burden is measured, ensuring that nonsalary components are included in performance and reimbursement frameworks.

Reforming payment models to recognize administrative efficiency while ensuring compliance with necessary regulations may help hospitals manage these growing costs. For example, promoting incentive programs like those established under the HITECH Act could encourage the adoption of cost-saving technologies, such as revenue cycle management and claims processing tools. Financial incentives have encouraged healthcare providers to transition to more efficient, automated systems, ultimately improving operational efficiency and healthcare delivery.^23^

#### Reducing payer-driven administrative complexity

Hospital executives have identified payer complexity as a source of increased administrative costs. The fragmented, multipayer system, with inconsistent billing and reimbursement, strains operations. Standardizing administrative processes across payer systems could help alleviate inefficiencies that drive high A&G costs.^24^ Legislative reforms should standardize contracts, streamline claims adjudication, and enhance transparency to reduce redundancies, disputes, and administrative waste, ultimately easing the burden on hospitals.^10^

This mixed-methods study highlights the evolving structure of hospital administrative spending. While administrative salaries are not increasing as a share of hospital expenses, rural hospitals continue to face disproportionate burdens and the overall growth in nonsalary administrative costs is accelerating. Addressing these challenges will require smarter, targeted policy responses that improve efficiency, reduce complexity, and support the unique needs of hospitals operating with limited resources.

### Future research

This study highlights important trends in A&G salary and total administrative spending across urban and rural hospitals, but additional questions remain. Further analysis is needed to explore how hospital size (eg, small community hospitals, CAHs, large academic medical centers, or part of a health system) influences administrative spending. Understanding whether larger hospitals achieve economies of scale in A&G expenditures could inform hospital consolidation strategies and operational restructuring efforts.

## Conclusion

Our analysis of Cost Report data from 2011 to 2022 reveals a shift in hospital spending patterns. Understanding how administrative spending is evolving is essential for strengthening hospital sustainability. While overall hospital costs and total A&G expenses have risen, administrative salaries as a proportion of total hospital spending have declined across all hospital types. The findings challenge prevailing narratives that place administrative salaries at the center of healthcare cost growth and instead highlight a broader reallocation of administrative resources, particularly in rural hospitals with fewer operational advantages.

Despite rising healthcare costs and the complexity of healthcare delivery, administrators represent a smaller share of hospital spending across all hospital types. Reported administrator's salaries as a proportion of total hospital costs have not increased in cost but rather decreased. At the same time, hospitals are redirecting more administrative spending toward nonsalary administrative costs. Addressing the financial force of administrative expenses requires targeted interventions to improve efficiency and sustainability. As healthcare evolves, continued research and policy attention are necessary to ensure that efforts to control administrative costs support, rather than compromise, hospital sustainability and the delivery of high-quality patient care.

## Supplementary Material

qxaf149_Supplementary_Data
