# Supplementary material for: Trends in hospital administrative costs: urban–rural disparities, barriers, and reduction strategies
Source: Health Aff Sch. 2025 Aug 8;3(8):qxaf149. doi: 10.1093/haschl/qxaf149 (PMC12359134; doi:10.1093/haschl/qxaf149)
Supplement: qxaf149_Supplementary_Data [file qxaf149_supplementary_data.zip › Supplemental Material.docx]

**Appendix A. Semi-Structured Interview Question Guide**


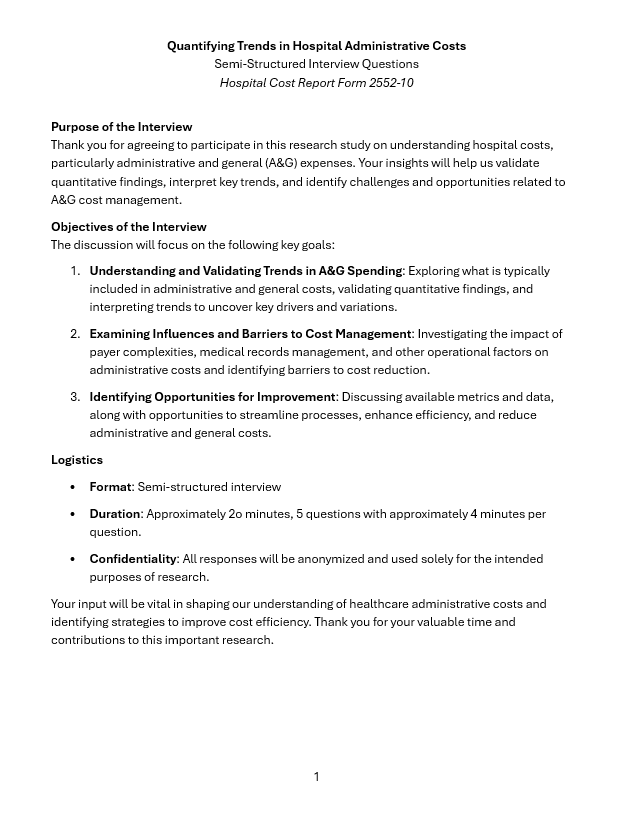


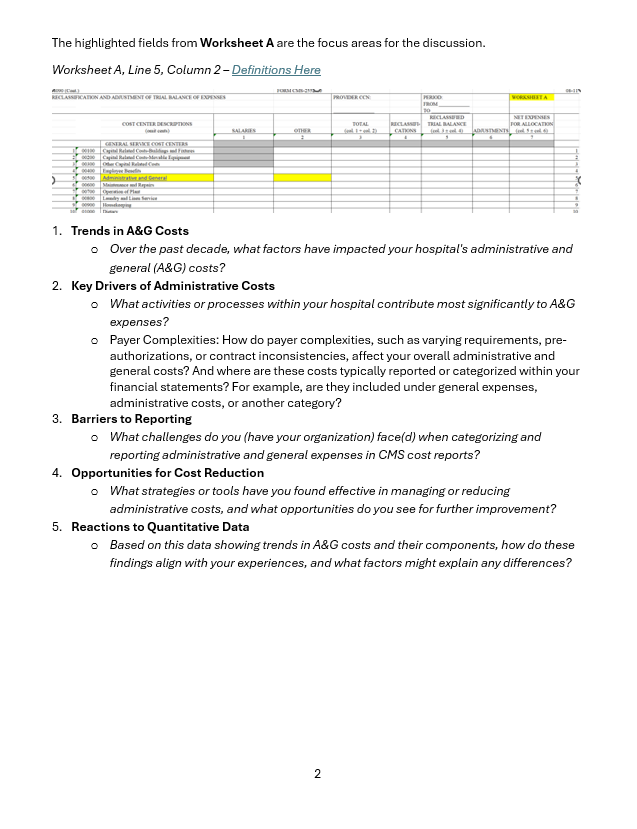


**Appendix B. Unadjusted Average Annual Total A&G Expenses as a Percent of Total Expenses, by Rurality, 2011 – 2022**


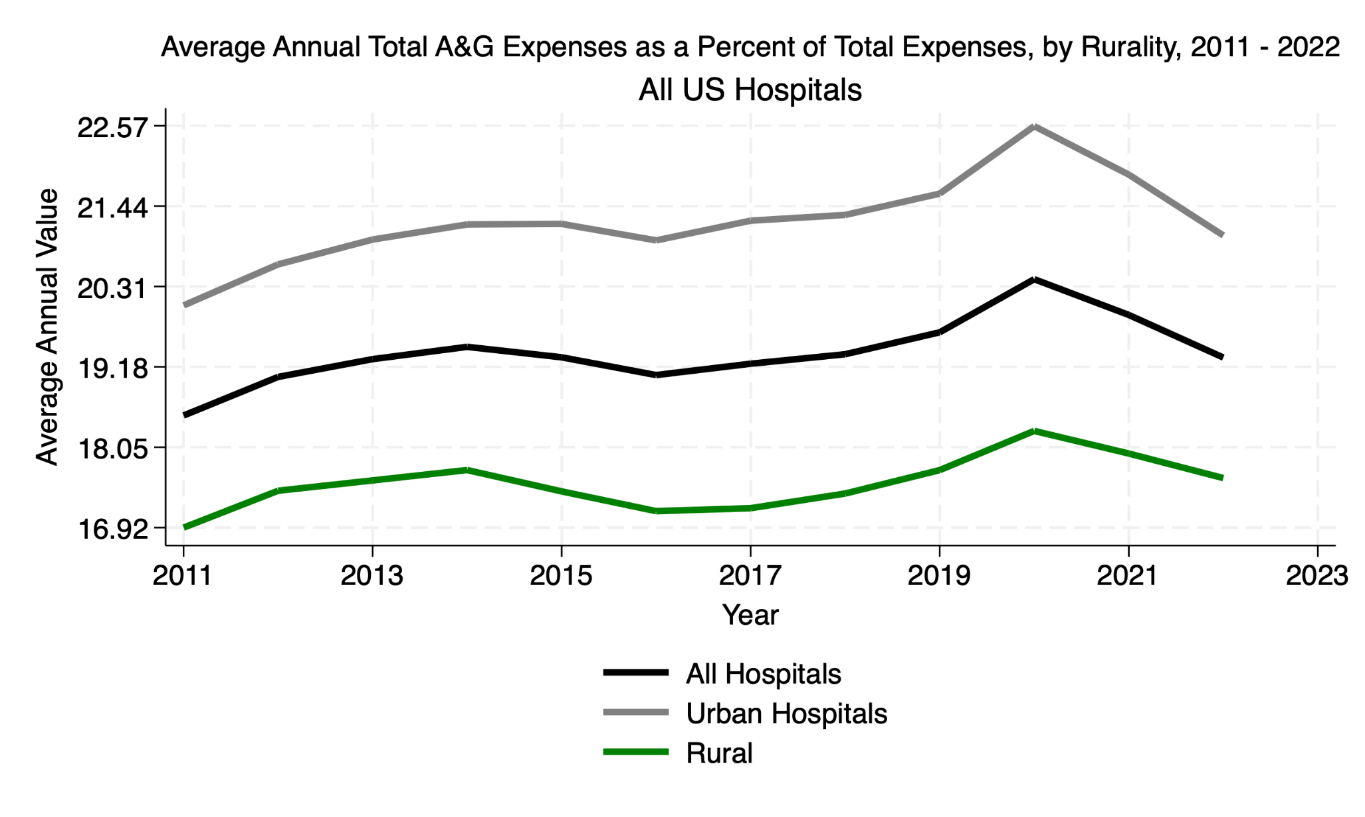


**Appendix C. Adjusted Total A&G as a Percent of Total Expenses, by Rurality, 2011 – 2022**


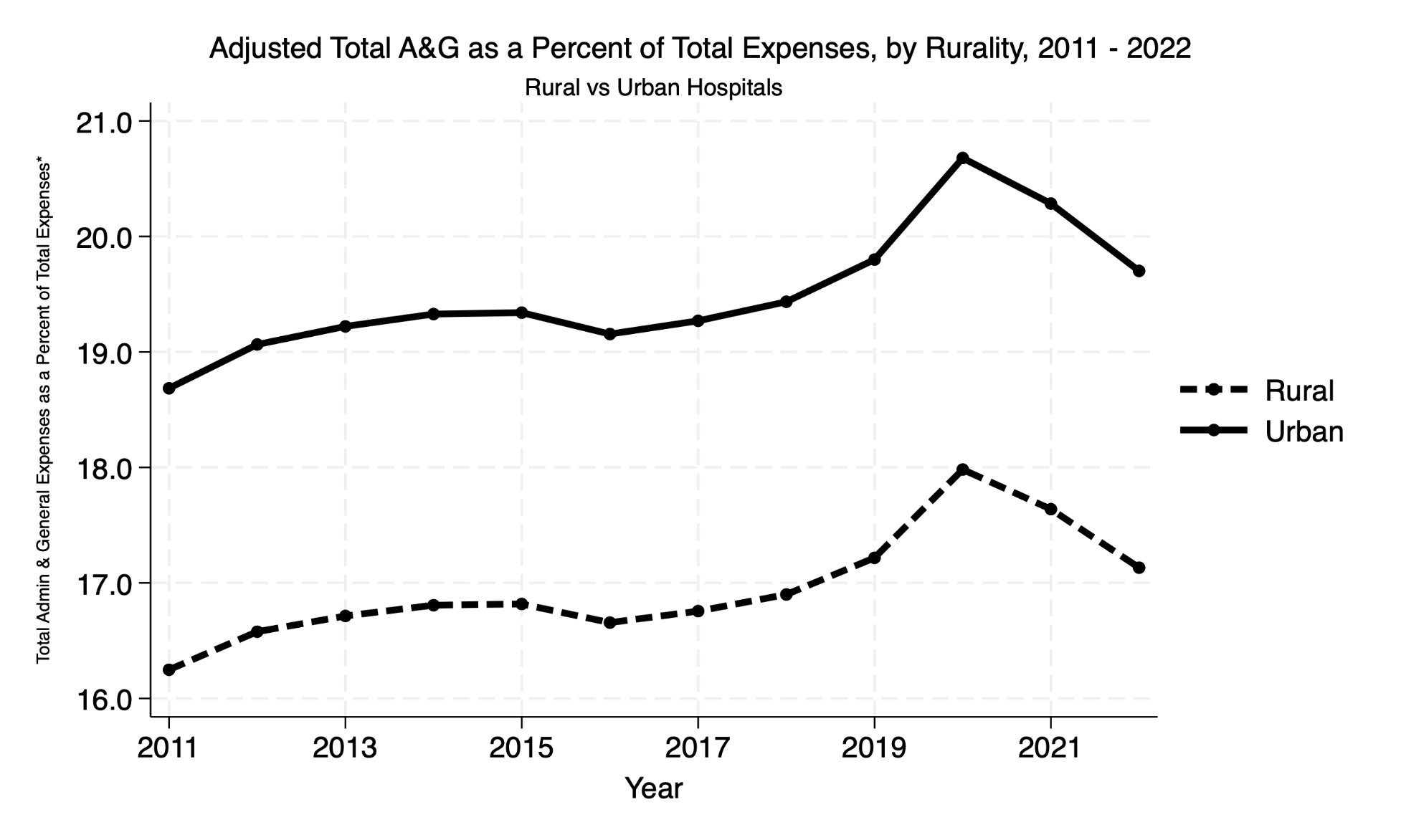


**Appendix D. Qualitative Domains and Subdomains**

| **Domains** | **Subdomains** |
| --- | --- |
| Trends in Administrative and General (A&G) Costs | Growth in A&G Costs Over Time |
|  | Changing Composition of A&G Expenses |
| Key Drivers of Rising A&G Costs | Expanding Regulatory and Payer Requirements |
|  | Revenue Cycle Management (RCM) Complexity |
|  | Technology and IT Infrastructure Costs |
|  | Legal Fees and Compliance Costs |
|  | Emergence of New Administrative Roles |
| Barriers to Accurate A&G Cost Reporting | Complexity of Medicare Cost Reporting |
|  | Rural vs. Urban Cost Reporting Constraints |
|  | Inconsistency in Categorization of Costs |
| Strategies to Manage and Reduce A&G Costs | Leveraging AI and Automation |
|  | Outsourcing Non-Core Administrative Functions |
|  | Organizational Cost Optimization |
|  | Legislative Advocacy against Payer Inefficiencies |

**Appendix E. Qualitative Subdomain Descriptions and Representative Quotes**

| **Domain/Subdomain** | **Description** | **Quote** |
| --- | --- | --- |
| **Trends in Administrative and General (A&G) Costs** | | |
| **Growth in A&G Costs Over Time** | A&G expenses have steadily increased as a proportion of total hospital costs, with notable differences in urban and rural cost growth, while A&G salary expenses have followed a diverging trend relative to total expenditures. | *"We've seen administrative expenses increase year over year, largely because of the growing complexity of hospital operations and regulatory requirements."* |
| **Changing Composition of A&G Expenses** | The composition of A&G expenses has shifted, with salaries making up a smaller proportion over time, while non-salary costs—including legal fees, consulting services, compliance-related expenses, and IT investments—have become significant contributors to overall administrative spending. | *"It’s not just salaries driving [A&G] costs anymore—consulting fees, legal expenses, and compliance-related costs have become a much bigger piece of the pie."* |
| **Key Drivers of Rising A&G Costs** | | |
| **Expanding Regulatory and Payer Requirements** | Increasing federal, state, and local regulations, along with evolving payer requirements, have significantly expanded administrative costs, as hospitals must allocate more resources to meet compliance, quality reporting, and reimbursement mandates. | *"The external requirements in various areas are driving [A&G] costs the most—whether it's quality metrics, safety mandates, or reporting regulations at the federal, state, or local level."* |
| **Revenue Cycle Management (RCM) Complexity** | The growing complexity of revenue cycle management, driven by rising claim denials, pre-authorization requirements, and payment disputes, has forced hospitals to invest in additional staff, specialized teams, and advanced billing technologies, contributing to increased A&G costs. Delays in payer reimbursements, prolonged claim disputes, and increasing levels of uncompensated care have placed financial strain on hospitals, requiring larger administrative teams to manage collections and revenue recovery. | *"Payers are increasingly finding ways to deny claims, delay payments, or underpay us. It takes more administrative resources to keep up, which means more staff, more technology, and ultimately, higher costs."   "It can take us 36 to 48 months to settle claims, and even then, we don’t get everything we’re owed. That kind of delay puts a huge strain on hospital finances."* |
| **Technology and IT Infrastructure Costs** | Hospitals have made substantial financial commitments to implementing and maintaining electronic health record (EHR) systems, cybersecurity protections, and subscription-based IT services, which have significantly added to A&G expenditures over time. | *"We invested millions into Epic and other technology to improve efficiency, but those costs don’t go away. The maintenance, security, and compliance requirements keep adding to our overhead."* |
| **Legal Fees and Compliance Costs** | Hospitals face rising legal and compliance-related expenses, including malpractice settlements and regulatory compliance costs, which have become a major driver of administrative spending. | *"Between legal battles over reimbursement and malpractice claims, our legal expenses have skyrocketed. Compliance requirements only add to that burden."* |
| **Barriers to Accurate A&G Cost Reporting** | | |
| **Complexity of Medicare Cost Reporting** | Medicare cost reporting has become increasingly complex, requiring specialized financial expertise to ensure accuracy, prevent errors, and avoid compliance penalties, which disproportionately impact resource-constrained hospitals. | *"Medicare cost reports have become so complex that even minor errors can lead to audits or financial penalties. It requires expertise, and not every hospital has the resources for that."* |
| **Rural vs. Urban Cost Reporting Constraints** | Rural hospitals, often operating with fewer financial and administrative resources, face unique challenges in accurately reporting A&G costs, as staff frequently manage multiple roles and lack dedicated personnel for financial reporting. | *"Many rural hospitals can’t afford full-time financial specialists, so cost reporting falls on staff who wear multiple hats. That leads to inconsistencies in how expenses are classified."* |
| **Inconsistency in Categorization of Costs** | Variability in how hospitals classify and allocate A&G expenses creates inconsistencies in cost reporting, making cross-hospital comparisons and benchmarking difficult. Differences in accounting practices, expense allocation methods, and reporting structures lead to challenges in accurately assessing A&G spending and its impact on hospital operations. | *"One of the biggest challenges is that hospitals don’t categorize administrative costs the same way. What one hospital includes in administrative & general, another might allocate elsewhere, making it difficult to compare across institutions or benchmark effectively."* |
| **Strategies to Manage and Reduce A&G Costs** | | |
| **Leveraging AI and Automation** | The integration of artificial intelligence and automation in revenue cycle management, billing, and administrative workflows presents an opportunity to reduce manual labor costs and improve operational efficiency. | *"Revenue cycle is probably the biggest area where AI can help cut costs. If we can streamline preauthorizations and denials, we could reduce staffing needs and improve cash flow."* |
| **Outsourcing Non-Core Administrative Functions** | Hospitals have increasingly turned to outsourcing non-core administrative tasks, such as billing, collections, and coding, as a cost-saving strategy, while ensuring oversight to maintain service quality. | *"We've outsourced some of our billing and collections, and it has improved our efficiency while reducing overhead. But you have to carefully oversee it to make sure quality doesn't suffer."* |
| **Organizational Cost Optimization** | Hospitals are actively consolidating administrative roles, reducing redundancies, and implementing cost-containment strategies to improve operational efficiency while ensuring financial sustainability. | *"We are continuously scrutinizing our administrative expenses, consolidating roles where possible, and advocating for legislative changes that could reduce unnecessary payer burdens."* |
| **Legislative Advocacy against Payer Inefficiencies** | Hospital leaders are engaging in legislative advocacy efforts to push for reforms that reduce administrative burdens, such as streamlining prior authorizations, enforcing timely payment rules, and addressing systemic inefficiencies in payer reimbursement processes. | *"We’ve been actively pushing for policy changes to reduce the administrative burden from payers—whether it’s streamlining prior authorizations or enforcing timely payment rules. The current system forces us to dedicate too many resources to fighting denials and delays."* |

**Appendix F. Missing Observations (N), 2011 & 2022**

| **Variable** | **2011** | **2022** |
| --- | --- | --- |
| **Demographic Characteristics** |  |  |
| Hospital Beds - Total | 10 | 7 |
| Employee FTEs | 23 | 11 |
| Ownership Type | 0 | 0 |
| Critical Access Hospital (CAH) | 0 | 0 |
| Region | 0 | 0 |
| Health System | 0 | 0 |
| **Operational Characteristics** |  |  |
| Average Daily Census | 0 | 0 |
| Length of Stay | 109 | 200 |
| Case Mix Index | 1,343 | 1,368 |
| **Financial Characteristics** |  |  |
| Total Expenses | 6 | 5 |
| A&G Total Expenses | 93 | 55 |
| A&G Salary Expenses | 101 | 74 |
| A&G Expenses to Total Expenses | 93 | 59 |
| A&G Salary Expenses to Total Expenses | 101 | 75 |
| Operating Margin | 155 | 93 |
| Days in Net A/R | 175 | 210 |
| Commercial-to-Medicare RCR | 24 | 47 |
| Total UCC and Bad Debt | 75 | 72 |
| Days Cash on Hand | 1,133 | 1,332 |
| LT Deb-to-Cap | 1,126 | 1,045 |
